# An Emerging Role of *Proanthocyanidins* on Psoriasis: Evidence from a Psoriasis-Like Mouse Model

**DOI:** 10.1155/2022/5800586

**Published:** 2022-06-08

**Authors:** Yang Yang, Yangmeng Zhao, Rui Lai, Li Xian, Qirong Lei, Jixiang Xu, Menglu Guo, Dehai Xian, Jianqiao Zhong

**Affiliations:** ^1^Department of Dermatology, The Affiliated Hospital of Southwest Medical University, Luzhou 646000, China; ^2^Department of Emergency, The Affiliated Hospital of Southwest Medical University, Luzhou 646000, China; ^3^Department of Neurobiology, Southwest Medical University, Luzhou 646000, China

## Abstract

**Background:**

Psoriasis is an immune-mediated, chronic inflammatory disease, and genetic, immune, oxidative stress (OS), and environmental factors are all thought to contribute to its occurrence. Proanthocyanidins (PCs) are natural flavonoids consisting of catechins and epicatechins which have anti-inflammatory and anti-OS activities. PCs have been widely used to treat various diseases, but reports regarding psoriasis are rare.

**Objective:**

To investigate the therapeutic effect and potential mechanisms of action of PCs in a psoriasis-like mouse model.

**Methods:**

Thirty male BALB/c hairless mice were assigned to six groups (*n* = 5): normal, model, low-dose PCs, medium-dose PCs, high-dose PCs, and control groups. The final five groups were dorsally exposed to 5% imiquimod (IMQ) cream once a day for 6 consecutive days, while the normal group received no intervention. Following the first day of IMQ application, mice in the PC-treated group were dosed with different amounts of PCs daily by oral gavage for six days, whereas mice in the control group received normal saline in the same way. One week later, skin lesions were evaluated by the severity of scoring system based on *psoriasis area and severity index* (PASI), and pathological alterations were assessed by hematoxylin-eosin (HE) staining. Indicators of inflammation or OS, such as interleukin- (IL-) 17, IL-23, phosphorylated-phosphatidylinositol 3-kinase (p-PI3K), phosphorylated-signal transducer and activator of transcription 3 (p-STAT3), superoxide dismutase (SOD), glutathione (GSH), malondialdehyde (MDA), catalase (CAT), vascular endothelial growth factor (VEGF), inducible nitric oxide synthase (iNOS), reactive oxygen species (ROS), and heme oxygenase-1 (HO-1), were determined by ELISA, RT-PCR, western blot, and immunohistochemistry (IHC) analysis.

**Results:**

IMQ administration induced the formation of large dark red plaques with thickly layered scales on the dorsal skin of mice; nevertheless, the lesions were substantially alleviated by PC administration. Histopathological alterations were observed in both model and control groups with epidermal hyperkeratosis, granulosa layer thinning, acanthosis, downward extension of rete ridges, dermal papillae expansion, capillary hyperplasia, and infiltration by inflammatory cells around blood vessels. These pathological changes, however, were restored by a range of doses of PCs, high-dose PCs in particular. Different doses of PCs significantly lowered the spleen index, levels of inflammatory or oxidative proteins (IL-17, IL-23, MDA, ROS, p-PI3K, and p-STAT3), and the mRNA expression of *Il-17*, *Il-23*, *Vegf*, and *iNos*. Protein and mRNA levels of anti-OS and anti-inflammatory biomarkers, including SOD, CAT, GSH, and HO-1, greatly increased after PC treatment, especially at the highest dose.

**Conclusions:**

Our findings reveal that PCs ameliorate psoriasis-like symptoms, suppressing the inflammatory response and mitigating OS damage in an IMQ-induced psoriasis-like mouse model. These effects are probably related to the inactivation of STAT3 and PI3K and activation of HO-1 signaling.

## 1. Introduction

Psoriasis is an immune-mediated, chronic, relapsing inflammatory disease that primarily affects the skin and joints. It has a prevalence estimated at 0.51% to 11.43% worldwide [1]. In histopathology, psoriasis is characterized by hyperproliferation of keratinocytes (KC), epidermal hyperplasia, dermal angiogenesis, and infiltration of inflammatory cells [2, 3]. Psoriasis does not just affect the skin but has been linked to the high incidence of systemic disorders, such as diabetes, cardiovascular disease, gastrointestinal dysfunction, and chronic nephropathy [4–7]. In addition, psoriasis has a strong impact on psychological health with an increased morbidity from depression, anxiety, and suicide [8, 9]. Various treatment approaches have been developed, including immunosuppressive agents, retinoid derivatives, corticosteroids, vitamin D3 analogs, calcineurin inhibitors, biologics, and UVA/UVB irradiation. However, many of these therapies have transient curative effects and fail to prevent relapse. In combination with the frequent high cost of treatment and serious side effects, there are few satisfactory therapies for this condition. Accordingly, there is a pressing need for the exploration and development of a long-term safe and effective approach to psoriasis.

The etiology of psoriasis is likely to involve genetic, immune, oxidative stress (OS), and environmental factors. However, immune-mediated inflammation and OS are considered to be the crucial factors in its pathogenesis [2]. Indeed, psoriatic inflammation is closely related to OS [10, 11] with redox imbalances throughout the course of psoriasis and notable aberrations of OS biomarkers in patients. For example, some oxidative/nitrative species (malondialdehyde (MDA), nitric oxide (NO), and inducible nitric oxide synthase (iNos)) are increased, while antioxidant enzymes, such as superoxide dismutase (SOD) and glutathione (GSH), are decreased in psoriatic lesions or serum [12–17]. Endogenous and exogenous factors cause increased generation of reactive oxygen species (ROS) which initiate OS and activate T helper (Th)1/Th17 cells to stimulate release of inflammatory cytokines, including interleukin- (IL-) 17, IL-22, IL-23, tumor necrosis factor-alpha (TNF-*α*), and interferon-gamma (IFN-*γ*). A series of inflammatory/OS-related signals, such as phosphatidylinositol 3-kinase (PI3K), signal transducer and activator of transcription 3 (STAT3), and heme oxygenase-1 (HO-1), are subsequently triggered to promote KC proliferation and angiogenesis. These molecular and histopathological changes eventually create the typical clinical manifestations of psoriasis [2, 18–23]. Therefore, targeting of inflammation and OS would be required as an appropriate approach to psoriasis treatment.

Proanthocyanidins (PCs) are a group of natural flavonoids derived from grapes, apples, cranberries, and black currants which contain catechin and epicatechin and possess anti-inflammatory, antioxidant, antiangiogenic, immunomodulatory, and antiproliferative properties [24–26]. Owing to these multifunctional capacities, PCs have been suggested to ameliorate many inflammation and OS-related complaints [25, 27, 28]. Previous work has confirmed that PCs activated HO-1 to reduce the production of ROS, NO, and MDA and promoted the expression of SOD and glutathione peroxidase (GSH-Px) [29, 30]. The impact of these effects is to mitigate oxidative damage. PCs also reduce the secretion of inflammatory factors, such as IL-2, IL-6, IL-8, IL-17, IFN-*γ*, and TNF-*α*, to inhibit inflammatory responses [31, 32]. In multiple-experimental studies, PCs have been demonstrated to scavenge ROS through blocking the Janus kinase (JAK)/STAT signaling pathway, thus inhibiting cell proliferation/migration and inflammation aggression [33, 34]. An *in vitro* effect of PCs on suppressing psoriatic KC growth and proliferation has also been reported [35]. Besides, PCs have been found to have few side effects and to be generally safe, even for pregnant women, during clinical trials. However, the efficacy of PCs for treatment of psoriasis has rarely been studied. Since immune-mediated inflammation and OS are hallmarks of psoriasis, the anti-inflammatory, antioxidant, antiproliferative, antiangiogenic, and immunomodulatory activities of PCs would make them ideal candidates for treatment of this disorder. We have previously speculated on the potential utility of PCs for psoriasis [21]. The current study used an animal model of psoriasis to investigate the potential for PCs administration to ameliorate psoriatic symptoms.

## 2. Materials and Methods

### 2.1. Mice

Five-week-old male hairless BALB/C mice (Chengdu Dossy Experimental Animals Co., Ltd., Chengdu, China; Licence NO. SCXK Chuan 2015-030) were housed at 18-24°C (50%–70% humidity) under a 12 h light/12 h dark cycle and fed with access to water and food *ad libitum*. Animal experiments were carried out in the SPF animal laboratory at the Center of Experimental Animal of Southwest Medical University.

### 2.2. Medicine and Reagents

PC powder (>95% purity) was purchased from Beijing Solabo Science and Technology Co., Ltd. (Beijing, China) and dissolved in normal saline (0.9%). Imiquimod (IMQ) cream (5%) was obtained from Sichuan Mingxin Pharmaceutical Co., Ltd. (Chengdu, China). Assay kits for SOD, GSH, MDA, and CAT were procured from Nanjing Jiancheng Technology Co., Ltd. (Nanjing, China), and ROS, IL-17, and IL-23 assay kits from Andy Gene Biotechnology Co., Ltd. (Beijing, China). TRIzol RNA extraction kit came from Kapa, Wilmington, USA, and anti-HO-1, -p-STAT3, -p-PI3K, and -*β*-actin primary antibodies and IgG secondary antibody were all from Bioworld, USA.

### 2.3. Ethics Statement

The present experiment was approved by the Animal Experimental Ethics Management Committee of the affiliated hospital of Southwest Medical University (Permit Number: 20190908) and was performed under the Guide for the Care and Use of Laboratory Animals. Surgical operations were carried out under intraperitoneal pentobarbital anesthesia to guarantee animals suffered the least pain and discomfort.

### 2.4. Animal Experiments

#### 2.4.1. Grouping

Thirty hairless BALB/c mice were randomly divided into six groups (*n* = 5): normal, model (IMQ only), low-dose PCs (IMQ+12.5 mg/kg PCs), medium-dose PCs (IMQ+25 mg/kg PCs), high-dose PCs (IMQ+50 mg/kg PCs), and control (IMQ+normal saline, i.e., NS) groups.

#### 2.4.2. Preparation of Different-Dose PCs

Low-dose, medium-dose, and high-dose PCs were, respectively, calculated as 12.5 mg/kg, 25 mg/kg, and 50 mg/kg per mouse per day. PC powder was dissolved in NS in 0.3 mL aliquots.

#### 2.4.3. Gavage Administration of PCs in Psoriasis-Like Mice Models

Mice in all groups, except the normal, received topical application of 5% IMQ cream (62.5 mg) to the dorsal region over an area of about 3 × 4 cm for 6 consecutive days. After the first day, mice in the PC groups were given PCs of appropriate concentrations in 0.3 mL/mouse doses by gavage once a day for 6 days while external IMQ application continued. The control group received an equal volume of NS. The model group received only IMQ, and the normal group received no treatment. During this period, skin lesions were observed daily by the naked eye, and photographs were taken. On the seventh day, mice were sacrificed and specimens of dorsal skin, serum, and spleen were collected for histopathological examination, measurement of OS and inflammation-related factors, and analysis of the spleen index.

### 2.5. Visual Skin Evaluation

The *psoriasis area and severity index* (PASI) was used to score dorsal lesions based on the parameters of erythema, scaling, and thickness (Table 1). Each parameter was scored independently on a scale from 0 to 4, as follows: 0 = no clinical signs, 1 = slight clinical signs, 2 = moderate clinical signs, 3 = marked clinical signs, and 4 = very marked clinical signs. The total score indicated the severity of lesions.

### 2.6. Spleen Index Assay

Spleens were excised and weighed after sacrifice. The spleen index was calculated as follows: spleen index (mg/g) = spleen weight (mg)/mouse weight (g).

### 2.7. Skin Tissue Preparation for Histopathology

Samples of dorsal skin were fixed with 4% paraformaldehyde, embedded in paraffin, and sliced into 3 *μ*m sections for hematoxylin-eosin (H&E) staining. Epidermal thickness from the granular to the basal layer was estimated at three randomly chosen sites on each slice. Two distinct fields were chosen and photographed under the microscope for each site. Mean values (± SD) of epidermal thickness, calculated by two investigators independently, are presented.

### 2.8. Analysis of OS and Inflammation-Related Indicators in Mice Serum

SOD, GSH, MDA, ROS, CAT, IL-17, and IL-23 were measured by enzyme-linked immunosorbent assay (ELISA) kits, according to the manufacturer's instructions. Optical density (OD) was read by Microplate Reader (Thermo Fisher Scientific, USA) and calculated from standard curves.

### 2.9. Western Blotting Analysis

Phosphorylated-PI3K (p-PI3K), p-STAT3, and HO-1 proteins were measured by western blotting. Samples of skin tissue were cut into small pieces and treated with lysate and protein loading buffers to extract total protein. Total protein concentration was measured by Pierce bicinchoninic acid (BCA) assay. Extracted proteins were subjected to tris-glycine sodium dodecyl sulfate polyacrylamide gel electrophoresis (SDS-PAGE) and electrotransferred to polyvinylidene difluoride membranes. Membranes were blocked with 5% skimmed milk in tris-buffered saline and tween (TBST) and incubated with primary antibodies, anti-p-PI3K (1 : 500), anti-p-STAT3 (1 : 500), and anti-HO-1 (1 : 500) at 4°C overnight followed by secondary antibody (horseradish peroxidase-labeled goat anti-rabbit IgG, 1 : 10000). Signals were detected by enhanced chemiluminescence system (Bio-Rad Laboratories, Inc., CA, USA) and analyzed by image software.

### 2.10. qRT-PCR (Real-Time Quantitative Reverse Transcription PCR) Analysis

Total RNA was extracted from skin samples by TRIzol RNA extraction kit, and mRNAs encoding OS-related genes, *Il-17*, *Il-23*, *Vegf*, *iNos*, *Ho-1*, *Cat*, *Sod*, and *Gsh* were detected according to the manufacturer's instructions using primers shown in Table 2. Target gene expression was determined relative to that of the internal control, *Gapdh*.

### 2.11. Immunohistochemistry (IHC) Analysis

Paraffin sections were dried at 65°C for 2 h, dewaxed, and subjected to microwave repair in EDTA buffer. Sections were incubated in 3% hydrogen peroxide solution at room temperature in the dark and blocked with 5% BSA. The BSA solution was removed, and 50 *μ*l of diluted primary antibody (1 : 200) was added for overnight incubation at 4°C. After washing, 100 *μ*l secondary antibody (1 : 200) was added and sections incubated at 37°C. Subsequently, the sections were developed using DAB solution for observation under the microscope. Brown nuclear/cytoplasmic granules indicated the expression of p-PI3K and p-STAT3 and brown sediments in the cytoplasm that of HO-1. Positive staining cells were counted by two independent observers in a double-blind visual microscope evaluation, and percentages of positively staining cells were calculated.

### 2.12. Statistical Analysis

Statistical analysis was performed by SPSS 22.0 software. Data are presented as mean ± SD with comparisons between two groups made by Student's *t*-test and multiple group comparisons by least significant difference (LSD) analysis of variance (ANOVA). A value of *p* < 0.05 was considered to indicate statistical significance. Orange 8.5 and GraphPad Prism 8.0 software were used to analyze data plots.

## 3. Results

### 3.1. PCs Ameliorated Skin Appearance in the Psoriasis-Like Mouse Model

**Figure 1:**
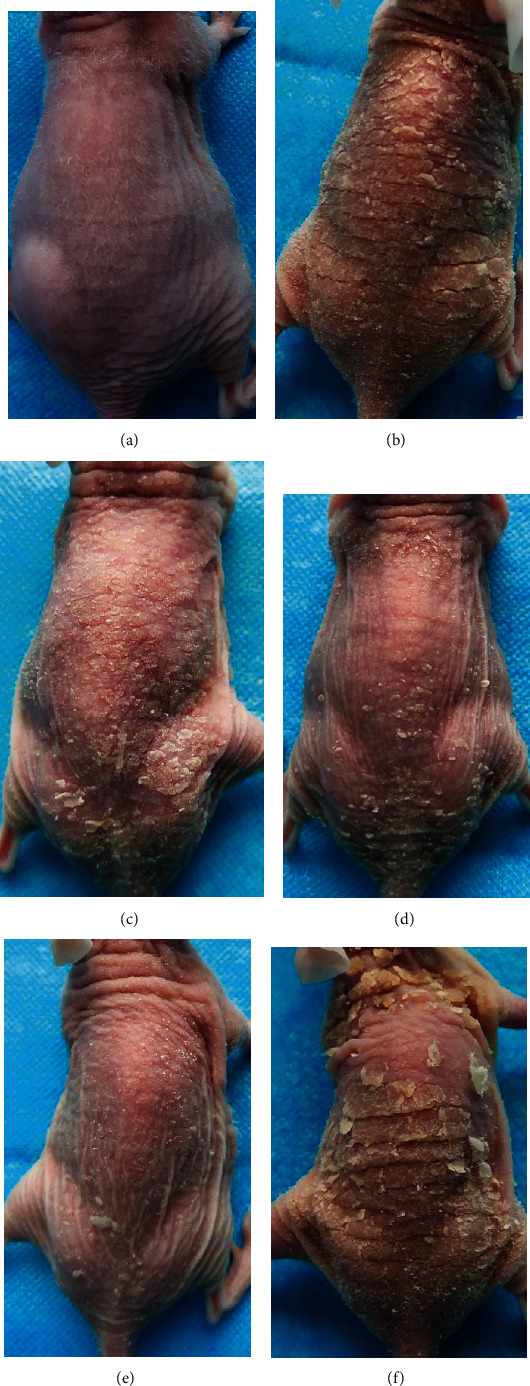
Skin visual manifestations of mice in different groups. (a) normal group; (b) model group, (c) low-dose PCs group, (d) medium-dose PCs group, (e) high-dose PCs group, (f) and control group.

**Figure 2:**
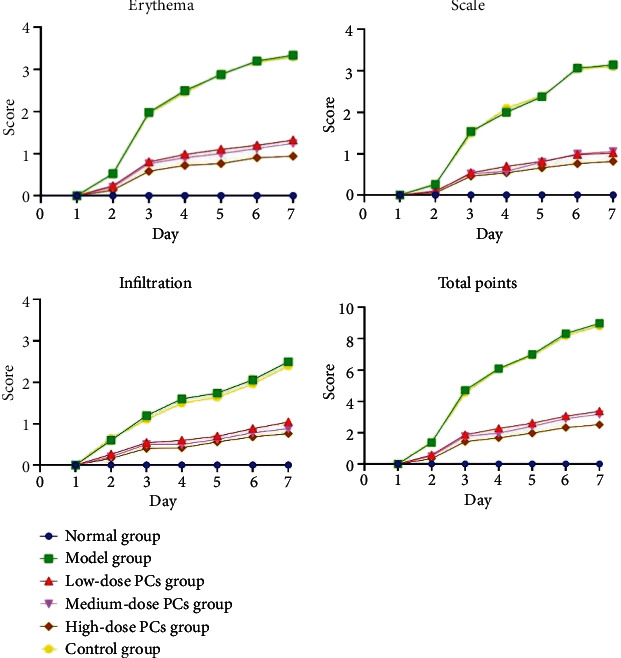
The severity scores of mice in different groups.

After seven days of IMQ application, psoriasis-like lesions, including swelling and extensive dark red plaques overlaid with silvery-white thick scales, could be visualized on dorsal skin, with severity scores of 8-10 (*p* < 0.05; Figures 1(a) and 1(b)). Lesions were relieved by PC treatment and showed ameliorated severity scores and visual appearance compared with the model group (*p* < 0.05; Figures 1 and 2). No significant improvements were observed in the control mice, regardless of skin manifestations or the severity scores (*p* > 0.05; Figures 1 and 2). Furthermore, all PC-treated groups had lower PASI scores than the control, especially in the high-dose group (*p* < 0.05; Figure 2).

### 3.2. PCs Alleviated Skin Histopathology in the Psoriasis-Like Mouse Model

**Figure 3:**
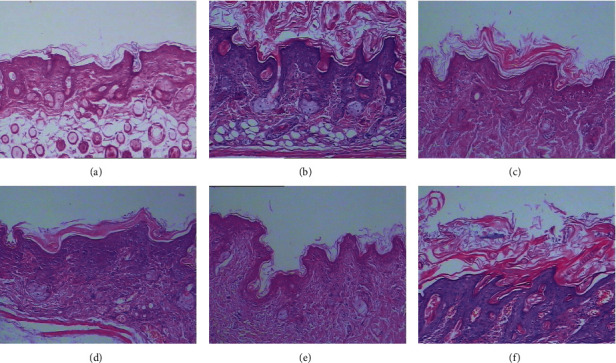
Histopathological changes of each group mice's back skin by HE staining (×100). (a) Normal group, (b) model group, (c) low-dose PC group, (d) medium-dose PC group, (e) high-dose PC group, and (f) control group. In (b) and (f), black arrows indicate that the epidermis became thick with parakeratosis/hyperkeratosis, granular layer deficiency, spinous layer hypertrophy, telangiectasia, and perivascular inflammatory cell infiltration in the dermis. In (c), (d), and (e), the above pathological changes were improved compared with (b) and (f), especially in (e).

Histological examination of normal mice showed a thin, 2-3-layer, epidermis without inflammatory cell infiltration of the dermis. After 7 days of IMQ administration, the epidermis thickened to 7-8 layers with parakeratosis/hyperkeratosis, granular layer deficiency, spinous layer hypertrophy and downward extension of epidermal rete ridges, dermal papilla stretching into the epidermis, telangiectasia, and perivascular inflammatory cell infiltration of the dermis. Such histopathological changes were similar to those observed in human psoriasis. PC treatment, however, notably ameliorated these changes, the high-dose PCs in particular. Little improvement was seen in the control group which was histopathologically similar to the model group (Figure 3).

### 3.3. PCs Reduced the Spleen Index in the Psoriasis-Like Mouse Model

IMQ application produced an increased spleen index, which was apparently reduced by PC administration relative to both the control and model groups, among which the high-dose group was the most prominent (*p* < 0.05; Table 3). No reduction of spleen index was observed in the control group (Table 3).

### 3.4. PCs Regulated Expression of Inflammatory and OS-Related Factors in the Psoriasis-Like Mouse Model

After 7-day application of IMQ, serum levels of OS and inflammatory factors, including IL-17, IL-23, MDA, and ROS, were dramatically elevated in the model group (*p* < 0.05), and those of antioxidant enzymes, SOD, CAT, and GSH, dropped (*p* < 0.05). PC treatment decreased the levels of IL-17, IL-23, MDA, and ROS (*p* < 0.05) and increased those of SOD, CAT, and GSH (*p* < 0.05). No statistically significant differences were observed in the control group relative to the model group (*p* > 0.05; Table 4).

### 3.5. PCs Affected Expression of Inflammation and OS-Related Genes in the Psoriasis-Like Mouse Model

**Figure 4:**
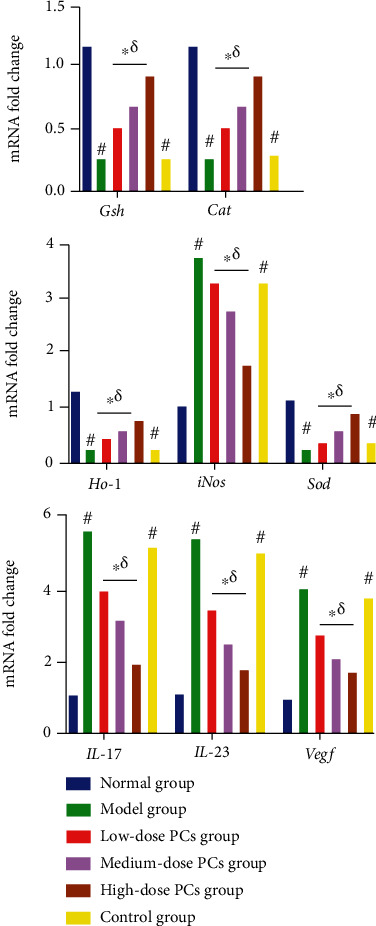
Relative expression of OS/inflammation-related genes in different groups. Note: compared with the normal group, ^#^*p* < 0.05; compared with the model group, ^∗^*p* < 0.05; and compared with the control group, ^*δ*^*p* < 0.05.

In comparison with the normal group, the model group showed elevated expression of *Il-17*, *Il-23*, *Vegf*, and *iNos* mRNA (*p* < 0.05) and suppressed expression of *Ho-1*, *Sod*, *Cat*, and *Gsh* mRNA (*p* < 0.05; Figure 4). By contrast, administration of PCs resulted in lower mRNA levels of *Il-17*, *Il-23*, *Vegf*, and *iNos* and higher mRNA levels of *Ho-1*, *Sod*, *Cat*, and *Gsh* (*p* < 0.05; Figure 4). The control group exhibited few changes relative to the model group (*p* > 0.05; Figure 4).

### 3.6. PCs Upregulated HO-1 and Downregulated p-PI3K and p-STAT3 Protein Expression in the Psoriasis-Like Mouse Model

**Figure 5:**
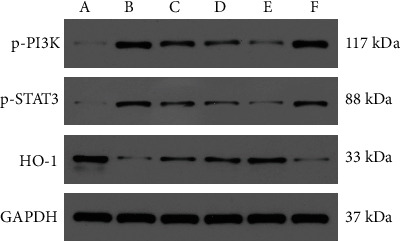
Effect of PCs on the expression of p-PI3K, p-STAT3, and HO-1 in the psoriasis-like mice by western blot analysis. Note: (a) normal group; (b) model group, (c) low-dose PC group, (d) medium-dose PC group, (e) high-dose PCs group, and (f) control group.

**Figure 6:**
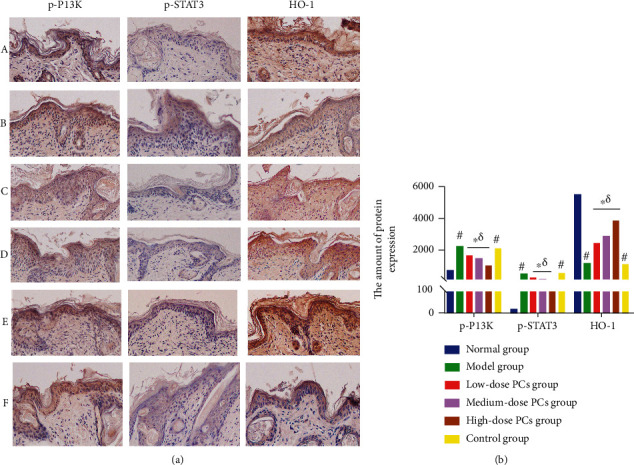
IHC analysis of p-PI3K, p-STAT3, and HO-1 expression in the psoriasis-like mouse. (a) Expression of p-PI3K, p-STAT3 and HO-1 in different groups. (A) normal group, (B) model group, (C) low-dose PC group, (D) medium-dose PC group, (E) high-dose PC group, and (F) control group. p-PI3K- and p-STAT3-positive cells show brown staining of the nucleus and cytoplasm; HO-1-positive cells show brown staining of the cytoplasm. Black arrows indicate p-PI3K-, p-STAT3-, or HO-1-positive cells. (b) Quantitative analysis of p-PI3K-, p-STAT3-, and HO-1-positive cells. Compared with the normal group, ^#^*p* < 0.05; compared with the model group, ^∗^*p* < 0.05; and compared with the control group, ^*δ*^*p* < 0.05.

Normal mice showed little expression of HO-1, p-PI3K, and p-STAT3 proteins in the skin. However, application of IMQ raised p-PI3K and p-STAT3 protein levels while decreasing that of HO-1 (*p* < 0.05; Figure 5). These changes were reversed by PC administration: HO-1 expression was enhanced, whereas that of p-PI3K and p-STAT3 declined, especially at the highest dose (*p* < 0.05; Figure 5). Similarly, insignificant differences were observed between the control group and the model group (Figure 5). IHC analysis showed that p-PI3K and p-STAT3 were rare in the epidermal cells of the normal group as brown granular nuclear/cytoplasmic staining, but p-PI3K-positive (p-PI3K+) cells and p-STAT3-positive (p-STAT3+) cells were apparently observed in the epidermis of the model group after IMQ application. However, PCs significantly lessened the p-PI3K+ cells and p-STAT3+ cells along with the enhancement of drug concentration (*p* < 0.05). The p-PI3K+ cells and p-STAT3+ cells in the control group were slightly different from those in the model group, but no statistical significance existed (*p* > 0.05). By contrary, HO-1 was evident in the epidermis as brown-stained sediments in the cytoplasm of the normal group; nevertheless, IMQ application greatly cut down the HO-1-positive (HO-1+) cells. However, the positive cells gradually went up with the increase of PC concentration. In similar results to those with the model group, minimal HO-1+ cells could be found in the control group (Figure 6).

## 4. Discussion

The current study reveals a novel impact of PCs on a mouse model of psoriasis *in vivo*. First, a psoriasis-like mouse model was successfully constructed through external use of IMQ. Then, skin symptoms, spleen index, and histopathological alterations were relieved by PC treatment, especially by the highest dose. Furthermore, PCs inhibited production of inflammatory mediators and regulated redox balance. Lastly, levels of PI3K/STAT3 were also reduced and those of HO-1 elevated in the presence of PCs. The above findings imply the capacity of PCs in mitigating inflammation and OS via inactivation of the PI3K/STAT3 pathway and activation of the HO-1 signal in order to alleviate experimental psoriasis.

Animal models of psoriasis have been constructed by a variety of approaches, including spontaneous production, genetic engineering, xenotransplantation, and direct induction, among which the last is frequently employed. The current study, therefore, adopted a direct induction method to establish a psoriasis-like mouse model through the application of IMQ. Since 2009, the IMQ-induced mouse model has been used extensively in the preclinical research of psoriasis [36]. Typical psoriasis-like lesions appeared in the dorsal region after IMQ administration, manifesting as thickened/swollen skin and dark red plaques covered with thick scales. The severity index based on PASI scores gradually increased, reaching 8-10 on the 6-7th day. In histopathology, it was presented as KC hyperproliferation, epidermal thickening with parakeratosis/hyperkeratosis, granular layer deficiency, spinous layer hypertrophy, dermal telangiectasia, and inflammatory cell infiltration. All these manifestations are analogous to those observed in human psoriasis. Spleen index increased as did proinflammatory marker expression (IL-17, IL-23, MDA, and ROS), whereas expression of redox regulators (SOD, CAT, and GSH) decreased in the psoriasis-like model. These changes were consistent with those previously reported in the literature [37–39]. Therefore, the successful generation of the psoriasis-like mouse model was confirmed.

PC doses of 12.5 mg/kg, 25 mg/kg, and 50 mg/kg by gavage were adopted, based on reports of the previous outcomes [40–42] and current preliminary experiments. Such a range of dosage is considered to reduce the likelihood of toxic side reactions. Skin lesions, erythema/scales, epidermal thickness, dyskeratosis/hyperkeratosis, vascular proliferation, and inflammatory cell infiltration were reduced in varying degrees by PC administration, and this was especially obvious in the high-dose group, as was the reduction of the spleen index. The control mice, which were given saline, showed few improvements and were similar to the model group. These findings were in agreement with those of Rauf et al., Katiyar, Long et al., Q. Li et al., and Jia et al. who found that PCs inhibited KC proliferation promoting normal epidermal keratinization and reduced angiogenesis and infiltration of inflammatory cells [25, 27, 28, 43, 44]. Thus, the initial observations from the current study indicate a potential role for PCs in the amelioration of psoriatic symptoms. Mechanisms of PC action were next addressed.

Many studies have shown an effect of PCs in a variety of diseases through downregulating expression of inflammatory and OS-related indicators and upregulating antioxidants. As the chief contributors to psoriasis pathogenesis, OS and the inflammatory response may be implicated in the mechanism of PC control of psoriasis. The current study found that IMQ produced increased levels of inflammatory and oxidative biomarkers (IL-17, IL-23, MDA, ROS, and VEGF) in skin and serum, while antioxidants (SOD, CAT, HO-1, and GSH) decreased. These findings indicate that IMQ application provoked a psoriasis-like inflammatory response and OS state. PC administration, however, reversed the above phenomena. Levels of proteins and mRNAs for IL-17, IL-23, MDA, VEGF, ROS, and iNOS descended, while those for SOD, CAT, HO-1, and GSH ascended in the presence of PCs. These effects may be closely relevant to the anti-inflammatory and antioxidant activities of PCs, which concurred with the reports of Lee et al., Sun et al., Chen et al., Zhang et al., and Bashir et al. [29–31, 45, 46] who observed that grape seed PCs lowered expression of MDA, IL-23, and TNF-*α* and heightened the activities of SOD, GSH, and HO-1. In summary, we conclude that PCs are likely to be efficacious in managing IMQ-induced psoriasis-like symptoms through preventing the inflammatory response and fighting OS damage. However, further mechanistic clarification is required.

Mounting evidence suggests the abnormal activation of many signaling pathways in psoriasis. Candidate pathways include MAPK (mitogen-activated protein kinase)/NF-*κ*B (the nuclear factor *κ*B) and PI3K/AKT and JAK/STAT [22, 47, 48] with the latter having particular significance for psoriasis [49–51]. Activation of PI3K and STAT3 promotes the excessive proliferation and abnormal differentiation of KC, infiltration of inflammatory cells, and secretion of cytokines (IL-6, IL-17, IL-22, and IL-23) in psoriasis [52–54]. These events culminate in the formation of inflammatory lesions in an IMQ-induced psoriasis-like model [55]. Thus, regulation of PI3K and STAT3 signals would inhibit the inflammatory response, heighten antioxidant activity, and reduce oxidative damage to tissues and cells [56, 57]. Suppression of PI3K/AKT/mTOR (mammalian target of rapamycin) signaling has also been suggested in the treatment of psoriasis [58] and the antioxidant protein, HO-1, may mitigate IMQ-induced psoriasis-like inflammation by blocking the STAT3 signal [59]. The aforementioned studies confirm the significance of PI3K, STAT3, and HO-1 in mediating the psoriatic state. The results from the current study show the stimulation of p-PI3K and p-STAT3 and suppression of HO-1 by IMQ application, changes which were reversed by administration of PCs and scarcely occurred in their absence. The indications are that PCs may alleviate psoriasis by blocking PI3K and STAT3-dominated inflammation/OS-related signals and stimulating HO-1 antioxidant signals to reduce inflammation and mitigate OS. Previous scholars have reported compatible findings. Bashir et al. found that inhibition of the PI3K/AKT-dependent pathway by PCs prevented DNA damage and enhanced HO-1 expression [46]. PCs have also been reported to activate HO-1 via prohibiting MAPK/NF-*κ*B signaling pathways to reduce the production of ROS, NO, and MDA, upregulate antioxidant activities (e.g., CAT, SOD, and GSH-Px), and arrest oxidative damage [31, 45]. Moreover, Zhang et al. and Song et al. demonstrated that PCs block JAK/STAT signaling to deplete ROS and reduce cell proliferation and migration as well as the progression of the inflammatory response [33, 34].

## 5. Conclusion

In summary, PCs relieve psoriasis-like symptoms, inhibiting the inflammatory response and attenuating oxidative damage, probably via inactivation of STAT3/PI3K and activation of HO-1. Further *in vitro* studies are planned to clarify mechanisms of PC action, including use of PI3K, STAT3, and HO-1 inhibitors. Our findings illuminate a promising psoriasis treatment and provide an experimental basis for mechanistic research into the antipsoriatic actions of PCs.

## Figures and Tables

**Table 1:** The severity index based on PASI of psoriatic mice models.

Score	0	1	2	3	4
Erythema	No erythema	Light red	Red	Dark red	Purplish red
Scale	No visible scale	Some lesions covered with tiny scales	Most lesions covered with patchy scales	Almost all lesions covered with thick scales	All lesions covered with thickly stratified scales
Infiltration	Lesions parallel to normal skin	Lesions slightly elevated above normal skin	Lesions moderately protuberant	Lesions obviously hypertrophic and protuberant	Lesions highly thickened and protuberant

**Table 2:** The primers of inflammation and OS-related genes.

Genes	Forward	Reverse
*Il-17*	5′-CTCAGACTACCTCAACCGTTCC-3′	5′-ATGTGGTGGTCCAGCTTTCC-3′
*Il-23*	5′-GGACTCAAGGACAACAGCCAG-3′	5′-AGGATCTTGGAACGGAGAAGG-3′
*Vegf*	5′-GCTACTGCCGTCCGATTGAG-3′	5′-GGCTTTGTTCTGTCTTTCTTTGGT-3′
*iNos*	5′-ACATCAGGTCGGCCATCACT-3′	5′-CAGAGGCAGCACATCAAAGC-3′
*Ho-1*	5′-CACAGATGGCGTCACTTCGT-3′	5′-TCCCTTACAGAGAGAAGGCCA-3′
*Sod*	5′-GAGACCTGGGCAATGTGACTG-3′	5′-CGCAATCCCAATCACTCCACA-3′
*Gsh*	5′-CCGTGGAAGATTTGAAGATGTC-3′	5′-CTGCTGCACCTTCTTAGTCCC-3′
*Cat*	5′-TACCTGTGAACTGTCCCTACCG-3′	5′-GCGTTTCACATCTACAGCGC-3′
*Gapdh*	5′-TGAAGGGTGGAGCCAAAAG-3′	5′-AGTCTTCTGGGTGGCAGTGAT-3′

**Table 3:** Changes of spleen index of mice in different groups.

Groups	Number (*n*)	Spleen index (mg/g)
Normal group	5	5.46 ± 0.86
Model group	5	10.46 ± 0.51^#^
Low-dose PC group	5	7.51 ± 0.46^∗^^&^
Medium-dose PC group	5	6.44 ± 0.46^∗^^&^
High-dose PC group	5	5.54 ± 0.67^∗^^&^
Control group	5	10.38 ± 0.52^#^

Note: compared with the normal group, ^#^*p* < 0.05; compared with the model group, ^∗^*p* < 0.05; and compared with the control group, ^&^*p* < 0.05.

**Table 4:** Levels of OS/inflammation-related indicators in different group ($\overset{¯}{x}\pm s$).

Groups	IL-17 (pg/mL)	IL-23 (ng/L)	ROS (U/mL)	MDA (nmol/mL)	SOD (U/mL)	CAT (U/mL)	GSH (*μ*mol/L)
Normal group	11.25 ± 0.19	38.32 ± 0.37	311.36 ± 3.33	9.56 ± 0.48	24.52 ± 0.27	95.53 ± 0.38	61.49 ± 0.46
Model group	21.53 ± 0.96^#^	44.49 ± 0.83^#^	386.93 ± 6.87^#^	18.41 ± 0.60^#^	22.39 ± 0.26^#^	39.87 ± 0.44^#^	28.28 ± 0.33^#^
Low-dose PCs group	16.51 ± 1.06^∗^^&^	35.24 ± 0.28^∗^^&^	343.05 ± 5.96^∗^^&^	15.47 ± 0.42^∗^^&^	23.35 ± 0.33^∗^^&^	55.62 ± 0.31^∗^^&^	44.52 ± 1.00^∗^^&^
Medium-dose PCs group	10.53 ± 0.51^∗^^&^	37.32 ± 0.55^∗^^&^	336.57 ± 8.37^∗^^&^	12.63 ± 0.49^∗^^&^	23.64 ± 0.16^∗^^&^	62.66 ± 0.34^∗^^&^	57.40 ± 0.49^∗^^&^
High-dose PCs group	9.96 ± 0.43^∗^^&^	36.94 ± 0.49^∗^^&^	332.15 ± 1.44^∗^^&^	8.59 ± 0.45^∗^^&^	24.13 ± 0.92^∗^^&^	61.61 ± 0.24^∗^^&^	47.80 ± 0.88^∗^^&^
Control group	21.92 ± 0.89^#^	43.65 ± 0.56^#^	383.42 ± 9.62^#^	19.26 ± 0.59^#^	22.40 ± 0.26^#^	38.59 ± 0.63^#^	27.91 ± 0.25^#^

Note: compared with the normal group, ^#^*p* < 0.05; compared with the model group, ^∗^*p* < 0.05; and compared with the control group, ^&^*p* < 0.05.

## Data Availability

The data used to support the findings of this study are included within the article.
